# Synergistic regulation of fusion pore opening and dilation by SNARE and synaptotagmin-1

**DOI:** 10.1093/jmcb/mjae011

**Published:** 2024-03-05

**Authors:** Kaiju Li, Kaiyu Li, Jiaqi Fan, Xing Zhang, Chengyan Tao, Yijuan Xiang, Lele Cui, Hao Li, Minghan Li, Yanjing Zhang, Jia Geng, Ying Lai

**Affiliations:** National Clinical Research Center for Geriatrics, State Key Laboratory of Biotherapy and Collaborative Innovation Center of Biotherapy, West China Hospital, Sichuan University, Chengdu 610041, China; Department of Laboratory Medicine, State Key Laboratory of Biotherapy and Cancer Center, West China Hospital, Sichuan University and Collaborative Innovation Center, Chengdu 610041, China; National Clinical Research Center for Geriatrics, State Key Laboratory of Biotherapy and Collaborative Innovation Center of Biotherapy, West China Hospital, Sichuan University, Chengdu 610041, China; National Clinical Research Center for Geriatrics, State Key Laboratory of Biotherapy and Collaborative Innovation Center of Biotherapy, West China Hospital, Sichuan University, Chengdu 610041, China; National Clinical Research Center for Geriatrics, State Key Laboratory of Biotherapy and Collaborative Innovation Center of Biotherapy, West China Hospital, Sichuan University, Chengdu 610041, China; Department of Laboratory Medicine, State Key Laboratory of Biotherapy and Cancer Center, West China Hospital, Sichuan University and Collaborative Innovation Center, Chengdu 610041, China; National Clinical Research Center for Geriatrics, State Key Laboratory of Biotherapy and Collaborative Innovation Center of Biotherapy, West China Hospital, Sichuan University, Chengdu 610041, China; National Clinical Research Center for Geriatrics, State Key Laboratory of Biotherapy and Collaborative Innovation Center of Biotherapy, West China Hospital, Sichuan University, Chengdu 610041, China; National Clinical Research Center for Geriatrics, State Key Laboratory of Biotherapy and Collaborative Innovation Center of Biotherapy, West China Hospital, Sichuan University, Chengdu 610041, China; Department of Laboratory Medicine, State Key Laboratory of Biotherapy and Cancer Center, West China Hospital, Sichuan University and Collaborative Innovation Center, Chengdu 610041, China; Department of Laboratory Medicine, State Key Laboratory of Biotherapy and Cancer Center, West China Hospital, Sichuan University and Collaborative Innovation Center, Chengdu 610041, China; Department of Laboratory Medicine, State Key Laboratory of Biotherapy and Cancer Center, West China Hospital, Sichuan University and Collaborative Innovation Center, Chengdu 610041, China; Tianfu Jincheng Laboratory, City of Future Medicine, Chengdu 641400, China; National Clinical Research Center for Geriatrics, State Key Laboratory of Biotherapy and Collaborative Innovation Center of Biotherapy, West China Hospital, Sichuan University, Chengdu 610041, China

**Keywords:** SNARE, synaptotagmin-1, fusion pore, vesicle exocytosis

## Abstract

Fusion pore opening is a transient intermediate state of synaptic vesicle exocytosis, which is highly dynamic and precisely regulated by the soluble N-ethylmaleimide-sensitive factor attachment protein receptor (SNARE) complex and synaptotagmin-1 (Syt1). Yet, the regulatory mechanism is not fully understood. In this work, using single-channel membrane fusion electrophysiology, we determined that SNAREpins are important for driving fusion pore opening and dilation but incapable of regulating the dynamics. When Syt1 was added, the closing frequency of fusion pores significantly increased, while the radius of fusion pores mildly decreased. In response to Ca^2+^, SNARE/Syt1 greatly increased the radius of fusion pores and reduced their closing frequency. Moreover, the residue F349 in the C2B domain of Syt1, which mediates Syt1 oligomerization, was required for clamping fusion pore opening in the absence of Ca^2+^, probably by extending the distance between the two membranes. Finally, in Ca^2+^-triggered fusion, the primary interface between SNARE and Syt1 plays a critical role in stabilizing and dilating the fusion pore, while the polybasic region of Syt1 C2B domain has a mild effect on increasing the radius of the fusion pore. In summary, our results suggest that Syt1, SNARE, and the anionic membrane synergically orchestrate the dynamics of fusion pore opening in synaptic vesicle exocytosis.

## Introduction

Synaptic vesicle fusion is a precisely regulated process that results in neurotransmitter release and postsynaptic response during neurotransmission. In pre-synapse, vesicle fusion is orchestrated by the soluble N-ethylmaleimide-sensitive factor attachment protein receptor (SNARE) complex, synaptotagmin-1 (Syt1), and other regulators (Brose et al., 1992; Weber et al., 1998; Chapman, 2008). During the process of neurotransmitter release, the fusion pore is a vital transient intermediate through which the luminal content of synaptic vesicles connects to synaptic cleft (Breckenridge and Almers, 1987; Choi et al., 2003). The nascent fusion pore, composed of lipids and transmembrane domains of SNAREpins, is highly variable and precisely regulated (Bao et al., 2016, 2018). Its opening and dilation might determine the fate of synaptic vesicles, either full-collapse fusing with the plasma membrane or closing and recycling via a ‘kiss-and-run’ mechanism (Alabi and Tsien, 2013; Wu et al., 2014; Shin et al., 2018). SNARE and Syt1 are thought to regulate the size and stability of fusion pores (Bao et al., 2018; Das et al., 2020).

SNAREpins, including Syntaxin-1A (STX1A) and synaptosomal-associated protein of 25 kDa (SNAP-25) locating on the plasma membrane and vesicle-associated membrane protein 2 (VAMP-2) on synaptic vesicles (Südhof and Rothman, 2009; Jahn and Fasshauer, 2012; Brunger et al., 2018), drive synaptic vesicle fusion by utilizing the energy from the formation of the four-helical bundle of the SNARE complex (Sutton et al., 1998; Weber et al., 1998). The copy number of the SNARE complex may regulate the size and stability of the fusion pore (Bao et al., 2018). Previous studies revealed that one SNARE complex is capable of inducing the formation of fusion stalk or transient fusion pore opening, whereas at least three SNAREpins are required to dilate the fusion pore (Van Den Bogaart et al., 2010; Shi et al., 2012; Wu et al., 2021).

Syt1, as the Ca^2+^ sensor on synaptic vesicles, synchronizes Ca^2+^-evoked neurotransmitter release but inhibits spontaneous release (Brose et al., 1992; Chapman, 2008). In the absence of Ca^2+^, the Syt1–SNARE–membrane superstructure is formed via the primary interface and the polybasic region of Syt1 C2B domain (Fernandez et al., 2001; Zhou et al., 2015; Brunger et al., 2018). In addition, Syt1 may oligomerize around fusion sites via a key residue, F349, in the C2B domain to prevent Ca^2+^-independent fusion, as demonstrated by fluorescence imaging and electrophysiology (Wang et al., 2014; Tagliatti et al., 2020). Upon binding to Ca^2+^, the insertion of the Ca^2+^-binding loop of Syt1 into the anionic membrane induces a conformational change in the Syt1–SNARE–membrane superstructure, thereby triggering synchronized neurotransmitter release (Bhalla et al., 2006; Lai et al., 2013; Bai et al., 2016; Brunger et al., 2019; Voleti et al., 2020). In both the reconstituted Ca^2+^-dependent fusion systems and mouse hippocampal neuronal synapses, mutations at the primary interface significantly obstruct the evoked neurotransmitter release, whereas mutations that disrupt the polybasic region of Syt1 exhibited a milder effect (Zhou et al., 2015). Recent studies revealed that Syt1 is also involved in regulating fusion pore dynamics in the nanodisc (ND)–black lipid membrane (BLM)-based electrophysiological system (Das et al., 2020). However, the mechanism through which SNARE and Syt1 synergically control the dynamics of fusion pores remains unknown.

Since the opening of the fusion pore is short-lived and highly dynamic, we used the electrophysiological system based on ND–BLM (Das et al., 2020), along with single-molecule fluorescence imaging techniques, to investigate the roles of SNARE and/or Syt1 in fusion pore dynamics, which may provide a molecular explanation of how SNAREs and Syt1 cooperatively regulate the dynamics of fusion pore opening in neurotransmitter release.

## Results

### The copy number of the SNARE complex determines fusion pore size

**Figure 1:**
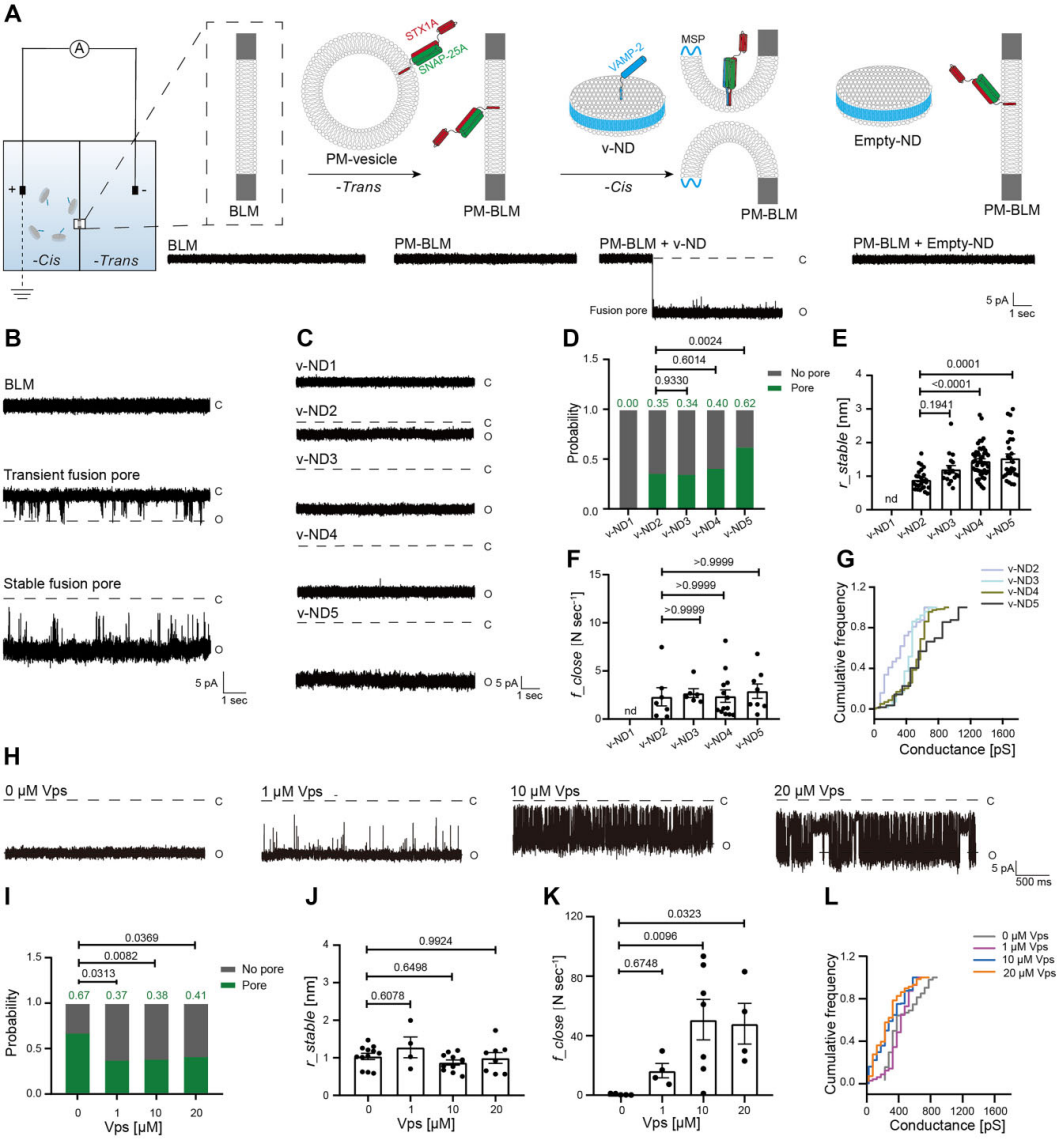
SNAREpins alone drive fusion pore opening. (**A**) Schematic illustration of the planar lipid bilayer electrophysiological assay of ND–BLM fusion. Fusion pores are formed between PM-BLM and v-ND under the voltage of −50 mV. (**B**) Typical traces of transient and stable fusion pores are shown, and closed (C) and open (O) states are indicated. (**C**) Typical traces of stable fusion pores for v-ND1, v-ND2, v-ND3, v-ND4, and v-ND5 are shown. (**D**) The probability of fusion pore formation. (**E**) The radius of stable fusion pores. (**F**) The closing frequency of stable fusion pores. (**G**) The distribution of cumulative single-channel conductance across different trials. (**H–L**) Vps, a cytosolic region of VAMP-2 (residues 1–96) was added at various concentrations. Typical traces of stable fusion pores for v-ND5 (**H**), the probability of fusion pore formation (**I**), and the radius (**J**), closing frequency (**K**), and representative cumulative conductance distribution (**L**) of stable fusion pores are shown. (**D** and **I**) The statistical analysis was performed using Pearson's χ^2^ test. (**E, F**, and **K**) Bar graphs represent mean ± SEM; Kruskal–Wallis test. (**J**) Mean ± SEM; one-way ANOVA.

To investigate the effects of the SNARE complex on fusion pore opening in a ND–BLM-based electrophysiological system, STX1A and SNAP-25A were reconstituted onto a liposome (referred to as PM-vesicle) and further deposited onto BLM to mimic the plasma membrane (referred to as PM-BLM), whereas VAMP-2 was reconstituted onto ND to mimic the synaptic vesicle membrane (referred to as v-ND) (Figure 1A; Supplementary Figure S1A–C). The diameters of v-NDs with various copy numbers of VAMP-2 were measured to be ∼13 nm by negative-stain transmission electron microscopy (TEM) (Supplementary Figure S1D and E). The copy numbers of VAMP-2 per ND were defined according to previous publications using the same reconstitution protocols and same lipid/protein ratios (Shi et al., 2013; Bao et al., 2016), and the copy number of VAMP-2 on v-ND5 was verified to be 7.11 per ND by the single-molecule photobleaching assay (Supplementary Figure S1F and G). When a voltage of −50 mV was applied, no current was detected from a stable BLM or PM-BLM, while a current change was observed at ∼−23 pA after the addition of v-ND, indicating a fusion pore formed at the PM-BLM (Figure 1A). However, the addition of empty-ND did not change the current (Figure 1A), suggesting that the fusion pore formation was SNARE-dependent. When the fusion pore was formed, the stable baseline of the current was defined as *I_0_*, where the amplitude of the blocking current upon closure was Δ*I*, and the current blockage rate was calculated as Δ*I*/*I_0_* (Supplementary Figure S2). The duration of the blocking current was defined as the ‘close dwell time’, and the interval between two blocking events was defined as the ‘open dwell time’ (Supplementary Figure S2). The closing frequency was evaluated as the number of occurrences of closure events within 1 sec. In addition to the stable open pores with a stable current baseline below 0 pA, some transient open pores with a baseline fluctuating around 0 pA were also observed (Figure 1B). This might represent a process that fusion pores close after rapid content release.

By changing the copy number of VAMP-2 onto ND, we found that it was positively correlated with the probability of fusion pore formation (Figure 1C and D). We also estimated the radius of stable open fusion pores from the measured conductance (Bao et al., 2018) and found that v-ND1 (likely one VAMP-2 molecule per ND) was not able to form fusion pores, while at least v-ND2 (perhaps two copies of the SNARE complex) was required to form fusion pores with an average radius of 0.893 nm (Figure 1C and E). Moreover, the increased copy number of VAMP-2 on ND resulted in the larger radius of fusion pores (Figure 1C and E), i.e. 1.205 nm for v-ND3, 1.449 nm for v-ND4, and 1.536 nm for v-ND5, respectively. In contrast, the closing frequency did not change with increasing copy number of VAMP-2 on ND (Figure 1C and F). The distribution of cumulative conductance across all trials confirmed that the copy number of the SNARE complex was critical for fusion pore size (Figure 1G). Consistently, increasing the copy number of the SNARE complex resulted in the higher fusion efficiency of content mixing (Supplementary Figure S3). However, we did not observe significant effects on the probability of transient fusion pore formation (Supplementary Figure S4A) or properties of transient fusion pores, i.e. the pore size and the opening frequency (Supplementary Figure S4B and C).

To verify that the fusion pore formation is SNARE-dependent, a cytosolic region of VAMP-2 (residues 1–96, Vps) was added at various concentrations to compete with v-ND, thereby leading to fusion pore closure (Figure 1H). Quantitative analyses revealed that Vps significantly inhibited the probability of fusion pore formation and cumulative conductance distribution (Figure 1I and L), significantly increased the closing frequency at >10 μm (Figure 1K), but did not affect the radius of fusion pores (Figure 1J). Moreover, when Vps was included, the Δ*I*/*I_0_* shifted from 0.22 to 0.78, the close dwell time increased from 0.39 ms to 1.09 ms, and the open dwell time decreased from 48.03 ms to 11.53 ms (Supplementary Figure S5). Taken together, SNAREpins alone were sufficient to drive the opening and expansion of fusion pores, but they might lack the ability to regulate the dynamics of fusion pore formation.

### Syt1/SNARE orchestrate the dynamics of fusion pore opening

**Figure 2:**
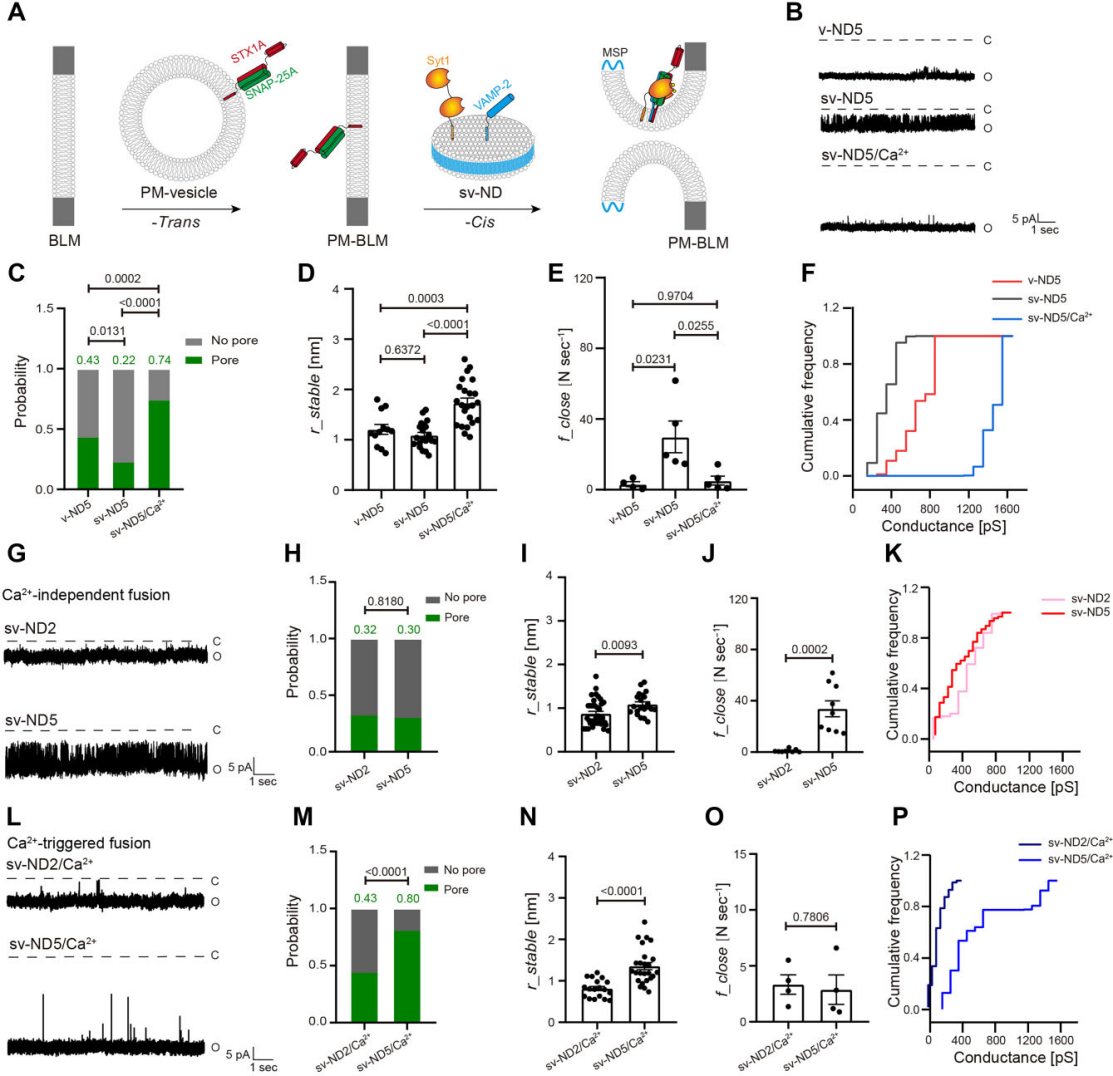
Syt1 and SNARE mediate fusion pore opening. (**A**) Schematic illustration of the planar lipid bilayer electrophysiological assay. (**B**–**F**) VAMP-2 only, VAMP-2/Syt1, or VAMP-2/Syt1 in the presence of Ca^2+^ were reconstituted onto ND. Typical traces of stable fusion pores (**B**), the probability of fusion pore formation (**C**), and the radius (**D**), closing frequency (**E**), and representative cumulative conductance distribution (**F**) of stable fusion pores are shown. (**G**–**K**) Different copies of VAMP-2/Syt1 were reconstituted onto ND in absence of Ca^2+^. Typical traces of stable fusion pores (**G**), the probability of fusion pore formation (**H**), and the radius (**I**), closing frequency (**J**), and representative cumulative conductance distribution (**K**) of stable fusion pores are shown. (**L**–**P**) Different copies of VAMP-2/Syt1 were reconstituted onto ND in the presence of Ca^2+^. Typical traces of stable fusion pores (**L**), the probability of fusion pore formation (**M**), and the radius (**N**), closing frequency (**O**), and representative cumulative conductance distribution (**P**) of stable fusion pores are show. (**C, H**, and **M**) Pearson's χ^2^ test. (**D** and **E**) Mean ± SEM; one-way ANOVA. (**I, J, N**, and **O**) Mean ± SEM; Student's *t*-test.

We next studied the effects of Syt1 on fusion pore properties by reconstituting VAMP-2 and Syt1, at the ratio of 4:1, onto ND to mimic the synaptic vesicle membrane (referred to as sv-ND) (Figure 2A and B). By negative-stain TEM and the size exclusion assay, we determined the purity of sv-ND and revealed that the average diameter of sv-ND was ∼13 nm (Supplementary Figure S6A–D), similar to that of v-ND. The copy number of Syt1 on sv-ND5 was measured to be 1.31 per ND by the single-molecule photobleaching assay (Supplementary Figure S6E and F). When Syt1 was included in the absence of Ca^2+^, the probability of fusion pore formation (from 0.43 to 0.22) and cumulative conductance distribution were inhibited, the closing frequency increased significantly from 3.18 N sec^−1^ to 29.88 N sec^−1^, but the radius of fusion pores remained unchanged (Figure 2C–F), suggesting that Syt1 has an inhibitory effect on Ca^2+^-independent fusion by destabilizing fusion pores. In the presence of 500 μM Ca^2+^, Syt1/Ca^2+^ significantly promoted the probability of fusion pore formation from 0.22 to 0.74 (Figure 2C), increased both the radius of fusion pores from 1.090 nm to 1.737 nm (Figure 2D) and the distribution of cumulative conductance (Figure 2F), and reduced the closing frequency from 29.88 N sec^−1^ to 5.16 N sec^−1^ (Figure 2E). Consistently, the inclusion of Syt1 in the absence of Ca^2+^ resulted in the Δ*I*/*I_0_* shift from 0.23 to 0.32, the close dwell time increase from 0.56 ms to 1.11 ms, and the open dwell time reduction from 73.74 ms to 4.95 ms (Supplementary  Figure S7A and B). In contrast, Syt1/Ca^2+^ prolonged the open dwell time from 73.74 ms to 178.21 ms but attenuated the Δ*I*/*I_0_* to 0.22 and shortened the close dwell time from 0.56 ms to 0.32 ms (Supplementary Figure S7A and C). We also found that the ratio of transient fusion pores decreased slightly in the presence of Syt1, particularly along with 500 μM Ca^2+^ (Supplementary Figure S8A and B), while neither the radius nor the opening frequency of transient fusion pores was affected (Supplementary Figure S8C and D). These findings suggested that Syt1/Ca^2+^ could facilitate SNARE-mediated fusion pore opening and dilation but was unable to regulate the formation of transient fusion pores, which might represent another route of SNARE-mediated neurotransmitter release, i.e. the ‘kiss-and-run’ pathway.

To further study the effect of the copy number of SNAREpins and Syt1 on fusion pore opening, we reconstituted sv-ND with lower copies of VAMP-2 and Syt1 at the ratio of 4:1 (referred to as sv-ND2) and compared it with sv-ND5 (Supplementary  Figure S6). In Ca^2+^-independent fusion, sv-ND2 resulted in the smaller radius lower closing frequency of fusion pores (Figure 2G, I, and J), but did not affect the probability of fusion pore formation and cumulative conductance distribution (Figure 2H and K). In the presence of 500 μM Ca^2+^, sv-ND2 significantly inhibited the probability of fusion pore formation, the radius of fusion pores, and the cumulative conductance distribution (Figure 2L–N and P) but slightly promoted the closing frequency (Figure 2O). Taken together, Syt1 inhibited fusion pore opening by increasing the closing frequency in the absence of Ca^2+^, whereas Syt1/Ca^2+^ could stabilize fusion pores and promote fusion pore dilation.

### The oligomer of Syt1 might be important for clamping fusion pore opening

**Figure 3:**
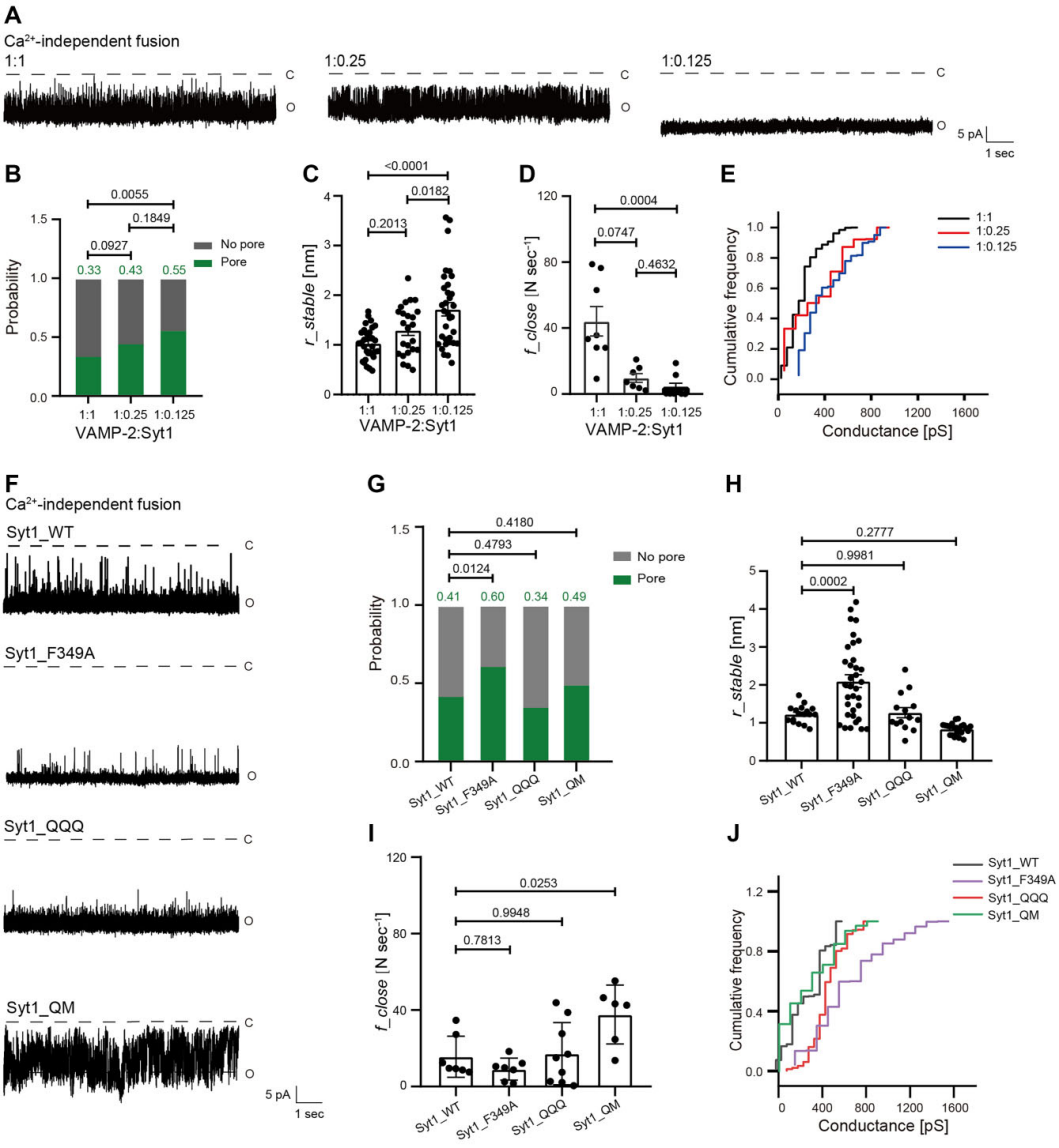
Syt1 clamps fusion pore opening in the absence of Ca^2+^. (**A**–**E**) VAMP-2/Syt1 at various ratios were reconstituted onto ND in the absence of Ca^2+^. Typical traces of stable fusion pores (**A**), the probability of fusion pore formation (**B**), and the radius (**C**), closing frequency (**D**), and representative cumulative conductance distribution (**E**) of stable fusion pores are shown. (**F**–**J**) VAMP-2 and wild-type or mutant Syt1 were reconstituted onto ND in absence of Ca^2+^. Typical traces of stable fusion pores (**F**), the probability of fusion pore formation (**G**), and the radius (**H**), closing frequency (**I**), and representative cumulative conductance distribution (**J**) of stable fusion pores are shown. (**B** and **G**) Pearson's χ^2^ test. (**C, H**, and **I**) Mean ± SEM; one-way ANOVA. (**D**) Mean ± SEM; Kruskal–Wallis test.

To investigate how Syt1 inhibits fusion pore opening, we reconstituted VAMP-2/Syt1 at ratios of 1:1, 1:0.25, and 1:0.125 onto ND and examined Ca^2+^-independent fusion pore opening (Figure 3A). The higher density of Syt1 on sv-ND5 led to the lower probability of fusion pore formation (Figure 3B), smaller radius of fusion pores (Figure 3C), higher closing frequency (Figure 3D), and lower cumulative conductivity (Figure 3E), indicating that the copy number of Syt1 was important for inhibiting Ca^2+^-independent fusion pore opening. We further examined several variants of Syt1: Syt1_F349A, which disrupts the region involved in Syt1 oligomerization (Tagliatti et al., 2020); Syt1_QQQ (K326/327/331Q), which mutates the polybasic region to block the interaction with the anionic membrane (Li et al., 2006; Loewen et al., 2006; Gaffaney et al., 2008); and Syt1_QM (quintuple mutant), which disrupts the primary interface between Syt1 and the SNARE complex (Zhou et al., 2015; Supplementary Figure S9). Compared to Syt1_WT in Ca^2+^-independent fusion pore opening (Figure 3F), Syt1_QM resulted in an obvious increase in the closing frequency (Figure 3I), whereas Syt1_QQQ had no significant effect (Figure 3G–J), suggesting that Syt1 binding to the SNARE complex or the anionic membrane was not a prerequisite for inhibiting fusion pore opening in the absence of Ca^2+^. Consistently, changing the concentration of phosphoserine (PS) on sv-ND had little effect on the radius or closing frequency of fusion pores (Supplementary Figure S10).

**Figure 4:**
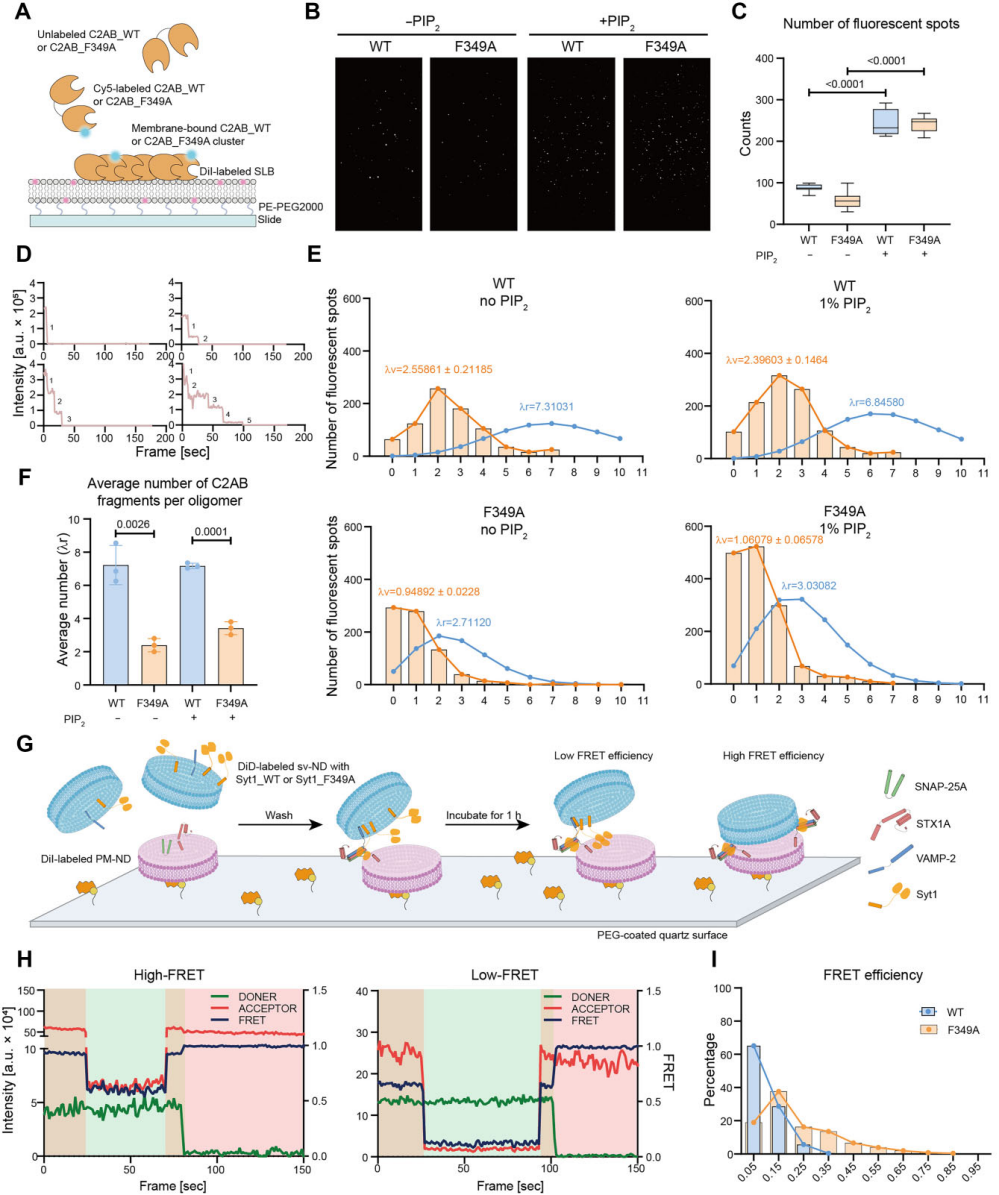
The oligomerization of Syt1 mediated by the residue F349 inhibits single-ND lipid mixing. (**A**–**F**) Single-molecule fluorescent photobleaching experiments determined Syt1 oligomerization and its membrane binding ability in the absence or presence of 1% PIP_2_. (**A**) Schematic illustration of Cy5-labeled C2AB clusters on DiI-labeled SLB. (**B**) Representative fields of view of the fluorescent spots corresponding to Cy5-labeled C2AB fragments binding to the SLB. (**C**) Box plot showing the number of fluorescent spots as shown in **B**. Error bars indicate standard deviations from >10 random imaging locations in the same sample channel. (**D**) Typical traces of single-molecule photobleaching steps. (**E**) Histograms of photobleaching steps observed for C2AB oligomers. The numbers of labeled C2AB fragments were fitted to Poisson distributions (orange lines), suggesting a random process for C2AB binding to the SLB. Blue lines are the calculated Poisson distributions of all (both labeled and unlabeled) C2AB fragments. (**F**) Bar graph showing the average number of C2AB fragments per fluorescent spot, which is the mean value (λr) calculated from the ‘real’ Poisson distributions (blue curves) in **E**. (**G**) Schematic diagram of the single-ND lipid mixing assay. DiI-labeled PM-ND is immobilized on the PEG-coated quartz surface to be allowed to interact with DiD-labeled sv-ND. (**H**) Representative traces of PM-ND and sv-ND fluorescence intensity and FRET efficiency. Orange background, both 532-nm and 635-nm excitation; green background, 532-nm excitation; red background, 635-nm excitation. (**I**) Histograms of FRET efficiency for Syt1_WT (blue)- or Syt1_F349A (orange)-mediated single-ND lipid mixing. (**C** and **F**) Mean ± SEM; Student's *t*-test.

Notably, Syt1_F349 was unable to clamp fusion pores (Figure 3F and H), although the closing frequency remained unchanged in the absence of Ca^2+^ (Figure 3I). Using the single-molecule fluorescent photobleaching assay with the Syt1 C2B domain-containing C2AB fragment (Figure 4A), we demonstrated that the F349A mutation did not interfere with the membrane binding of C2AB fragments in the absence or presence of 1% PIP_2_, although the inclusion of PIP_2_ significantly enhanced the membrane binding affinity of both wild-type (C2AB_WT) and C2AB_F349A fragments (Figure 4B and C). Moreover, in the presence of 1% PIP_2_, C2AB_WT formed oligomers on the supported lipid bilayer (SLB) with an average of 6.846 molecules, while C2AB_F349A only formed oligomers with an average of 3.031 molecules (Figure 4D–F). Notably, we did not observe a ring-like structure of C2AB in the presence of 1 mm adenosine 5′-triphosphate by negative-stain TEM (Supplementary Figure S11), and the inclusion of 1% PIP_2_ had little effect on the oligomerization of C2AB (Figure 4E and F), indicating that oligomerization of Syt1 did not rely on its binding to the membrane. Finally, to study the functional consequence of Syt1 oligomerization in fusion pore formation, we performed the single-ND lipid mixing assay by labeling PM-ND and sv-ND with the lipid fluorophores DiI and DiD, respectively (Figure 4G). When PM-ND and sv-ND were mixed, the fluorescence resonance energy transfer (FRET) efficiency for the lipid mixing mediated by Syt1_WT was significantly lower than that mediated by Syt1_F349A (Figure 4H and I). These findings suggested that the oligomer of Syt1 might play a role in inhibiting SNARE-mediated membrane contact by increasing the distance between two NDs, thereby preventing Ca^2+^-independent fusion pore opening.

### Syt1 promotes Ca^2+^-triggered fusion pore opening by interacting with the SNARE complex via the primary interface

**Figure 5:**
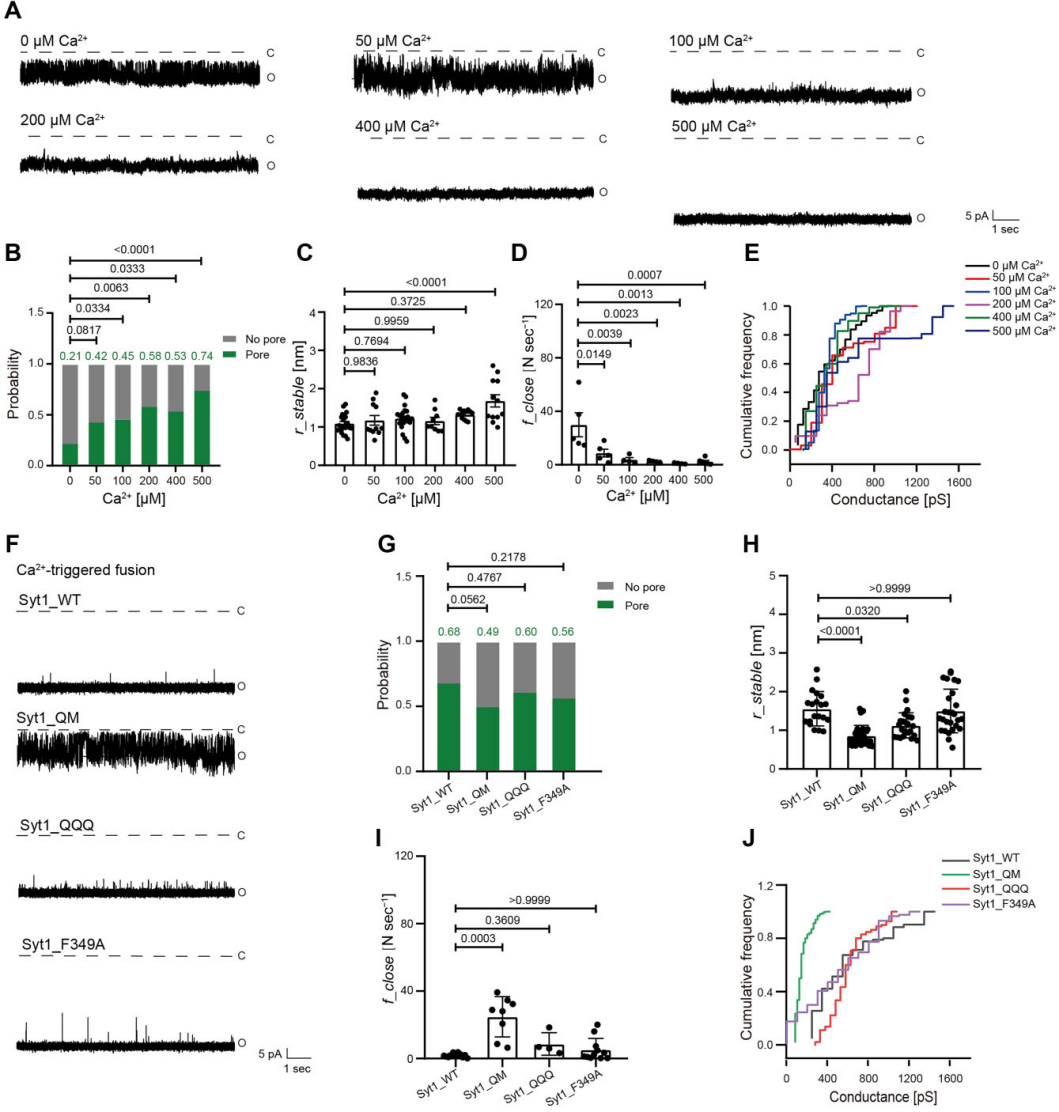
Syt1/Ca^2+^ promote and stabilize fusion pore opening. (**A**–**E**) VAMP-2/Syt1 were reconstituted onto ND in the presence of Ca^2+^ at various concentrations. Typical traces of stable fusion pores (**A**), the probability of fusion pore formation (**B**), and the radius (**C**), closing frequency (**D**), and representative cumulative conductance distribution (**E**) of stable fusion pores are shown. (**F**–**J**) VAMP-2 and wild-type or mutant Syt1 were reconstituted onto ND in presence of Ca^2+^. Typical traces of fusion pores (**F**), the probability of fusion pore formation (**G**), and the radius (**H**), closing frequency (**I**), and representative cumulative conductance distribution (**J**) of stable fusion pores are shown. (**B** and **G**) Pearson's χ^2^ test. (**C** and **D**) Mean ± SEM; one-way ANOVA. (**H** and **I**) Mean ± SEM; Kruskal–Wallis test.

Responding to Ca^2+^ influx upon action potential is a critical step for SNARE/Syt1 to trigger synchronized neurotransmitter release. Our results revealed that increasing Ca^2+^ concentration could greatly promote and stabilize SNARE/Syt1-mediated fusion pore opening by increasing the probability of fusion pore formation from 0.21 to 0.74 (Figure 5A and B), enlarging the radius of fusion pores from 1.090 nm to 1.681 nm (Figure 5C), increasing the distribution of cumulative conductance (Figure 5E), and reducing the closing frequency from 29.88 N sec^−1^ to 0.98 N sec^−1^ (Figure 5D). In addition, increasing the density of Syt1 on sv-ND5 did not affect the radius and closing frequency of fusion pores but mildly promoted the probability of fusion pore formation and cumulative conductivity (Supplementary Figure S12). Consistently, Syt1_F349A had little effect on Ca^2+^-triggered fusion pore opening (Figure 5F–J), indicating that the oligomerization of Syt1 was not necessary for Ca^2+^-triggered fusion pore opening.

However, the radius of fusion pores (from 1.56 nm to 0.86 nm) and the cumulative conductance distribution decreased markedly, while the closing frequency increased significantly from 1.92 N sec^−1^ to 24.84 N sec^−1^ when the primary interface was disrupted by Syt1_QM (Figure 5H–J), suggesting that Syt1 binding to the SNARE complex via the primary interface played a critical role in dilating and stabilizing the fusion pore in the presence of Ca^2+^. Syt1_QQQ also slightly reduced the radius of fusion pores (Figure 5H), without affecting the probability of fusion pore formation, the closing frequency, or cumulative conductivity (Figure 5G, I, and J), suggesting that Syt1 binding to the membrane via the polybasic region also played a role in dilating the fusion pore.

## Discussion

SNARE and Syt1 have been reported to be critical for regulating fusion pore opening (Bao et al., 2016, 2018; Das et al., 2020). However, the detailed mechanism is still unclear because of the highly dynamic and transient state of the fusion pore. In this work, we utilized single-channel membrane fusion electrophysiology to uncover the cooperative function of SNARE and Syt1 in regulating the size and dynamics of fusion pores. Previous studies showed that one SNAREpin could form fusion stalks, while at least three SNAREpins were needed to form fusion pores, leading to neurotransmitter release. We confirmed that the fusion pore size was determined by the copy number of SNAREpins, and at least two SNAREpins were required to drive fusion pore opening (Figure 1). However, changing the copy number of SNAREpins had little effect on the closing frequency of fusion pores (Figure 1F). These results indicated that SNARE might only function as an energy producer for opening and dilating fusion pores but was unable to regulate the dynamics.

Syt1 was reported to enhance the docking and priming of synaptic vesicles in reconstituted systems and cultured neurons (Kim et al., 2012; Imig et al., 2014); however, it was also observed to function as a fusion clamp to prevent spontaneous release in physiological experiments (Xu et al., 2009; Wang et al., 2011; Courtney et al., 2019). Using single-channel membrane fusion electrophysiology, we observed an inhibitory effect of Syt1 on Ca^2+^-independent fusion pore opening (Figure 2B–F). Specifically, the inclusion of Syt1 increased the close dwell time, the Δ*I*/*I_0_*, and the closing frequency but decreased the open dwell time and the radius of fusion pores (Figure 2D and E; Supplementary Figure S7A and B). Moreover, the copy number of Syt1 on ND was critical for inhibiting Ca^2+^-independent fusion pore opening by reducing the radius and closing frequency of fusion pores (Figure 3C and D). Previous studies reported that the cytosolic domain of Syt1 can assemble into a ring-like structure under Ca^2+^-free conditions, which could isolate the vesicle and plasma membranes, prevent *cis*-SNARE complex formation, and inhibit membrane fusion in the absence of Ca^2+^ (Wang et al., 2014). Although the formation of the ring-like structure was not detected by negative-stain TEM (Supplementary Figure S11), the oligomerization of C2AB_WT on the SLB in the absence of Ca^2+^ was observed by using single-molecule fluorescent photobleaching technology (Figure 4A and B). Consistently, F349A mutation disrupted C2AB oligomerization (Figure 4), which resulted in the larger radius of fusion pores (Figure 3H). Moreover, the oligomer cluster of Syt1 situated at the planar lipid bilayer hindered lipid mixing between the two NDs (Figure 4G–I), probably by serving as a cushion to increase the membrane distance and prevent lipid contact. Thus, our results demonstrated that the oligomer of Syt1 might play a pivotal role in clamping fusion pore opening in the absence of Ca^2+^. However, the interactions among Syt1, the SNARE complex, and anionic lipids seem not to directly contribute to this inhibitory effect on Ca^2+^-independent fusion pore opening, suggesting that Syt1 may utilize different molecular mechanisms to regulate Ca^2+^-independent and Ca^2+^-stimulated membrane fusion. Since complexin was omitted in our fusion system, we cannot rule out complexin additionally clamping Ca^2+^-independent fusion pore opening, for instance, via the tripartite interface between the SNARE complex, Syt1, and complexin (Zhou et al., 2017).

In the presence of Ca^2+^, Syt1 significantly increased the open dwell time and the radius of fusion pores but reduced the close dwell time, the Δ*I*/*I_0_*, and the closing frequency (Figure 2D and E; Supplementary Figure S7). The primary interface of Syt1 is critical for fast synchronous Ca^2+^-triggered neurotransmitter release in both cultured neurons and reconstitution systems (Zhou et al., 2015); however, how Syt1 implements this function has not been determined. Here, we found that the primary interface between Syt1 and the SNARE complex plays a pivotal role in stabilizing and dilating fusion pores in the presence of Ca^2+^ (Figure 5). Moreover, the polybasic region of Syt1, which interacts with the anionic membrane, also serves to dilate Ca^2+^-triggered fusion pores by increasing the radius (Figure 5). In addition, Ca^2+^-dependent membrane insertion of Syt1 could lower the free energy of membrane fusion, thereby facilitating pore dilation and cargo release (Wu et al., 2021). Thus, the supermolecular arrangement of SNARE, Syt1, and the membrane via the primary interface, the polybasic region, and the Ca^2+^-binding region of Syt1 might be important for opening and stabilizing Ca^2+^-triggered fusion pores.

In the trials under different conditions, transient fusion pores were commonly observed (Figure 1; Supplementary Figures S4 and S8). Given that the baseline of recording traces maintained at ∼0 pA and fusion pores rapidly opened for several milliseconds, we suspected it representing the unclassical ‘kiss-and-run’ pathway. We found that when Syt1 and Ca^2+^ were included, the fraction of transient fusion pores was reduced from 35% to <21% (Supplementary Figure S8B), suggesting that Syt1/Ca^2+^ tends to push vesicles to the full-collapse fusion pathway. However, the radius and opening frequency of transient fusion pores did not change (Supplementary Figure S8), indicating that SNARE and Syt1 do not regulate transient fusion pores. Recent studies revealed that Syt7 or other factors might determine the fate of vesicles toward the ‘kiss-and-run’ pathway (Alabi and Tsien, 2013; Wu et al., 2014; Shin et al., 2021). Future investigations are needed to uncover the mechanism regulating the size and dynamics of transient fusion pores.

In this study, we used an entirely *in-vitro* reconstitution system to mimic the physiological process of fusion pore opening during neurotransmitter release. The lipid composition and protein-to-lipid ratio were determined following the reported physiological conditions in the pre-synapse (Takamori et al., 2006). The findings on the molecular mechanism of SNARE-mediated vesicle exocytosis from such reconstitution experiments have been verified by physiological studies (Weber et al., 1998; Fernández-Chacón et al., 2001; Tucker et al., 2004; Zhou et al., 2015; Bao et al., 2018; Lai et al., 2022). Nonetheless, we admit that our results still merit further studies under physiological conditions.

**Figure 6:**
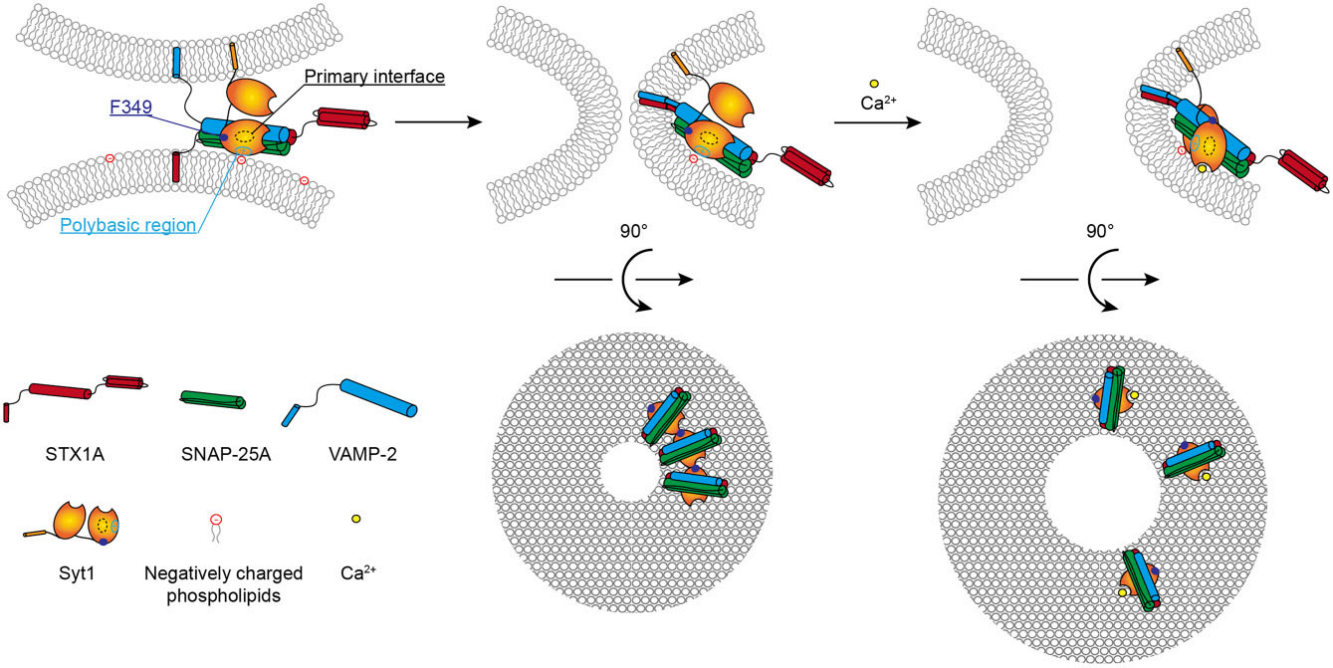
Working model of how SNARE and Syt1 synergistically regulate fusion pore opening. In the pre-fusion state, SNAREpins, including STX1A, SNAP-25A, and VAMP-2, together with Syt1 and the anionic membrane, form a supermolecular arrangement via the primary interface and the polybasic region of Syt1 C2B domain. In the absence of Ca^2+^, SNAREpins alone can drive the formation of fusion pores. However, Syt1 may restrict the opening and dilation of the fusion pore by its oligomerization via the residue F349. When Syt1 binds to Ca^2+^, the insertion of the Ca^2+^-binding loop of Syt1 into the membrane results in a rearrangement of the SNARE–Syt1–membrane superstructure, leading to the disruption of Syt1 oligomer and consequently the release of its clamping effect on fusion pore opening. During Ca^2+^-triggered synaptic vesicle fusion, the primary interface and the polybasic region of Syt1 C2B domain are important for stabilizing and dilating the fusion pore by mediating Syt1 interaction with the SNARE complex and anionic lipids, which ultimately results in the full-collapse fusion of synaptic vesicles with the plasma membrane.

In summary, we proposed a model in which Syt1 and SNARE synergically regulate fusion pore opening (Figure 6). In the absence of Ca^2+^, sufficient copies of SNAREpins can spontaneously drive fusion pore opening, but the formation of Syt1 oligomers prevents the opening and dilation of fusion pores, probably by extending the distance between two membranes. When Syt1 binds to Ca^2+^ and interacts with SNAREpins and the membrane, they form a supermolecular arrangement via the primary interface and the polybasic region of the Syt1 C2B domain, which is essential for stabilizing and dilating Ca^2+^-triggered fusion pores. Taken together, SNARE, Syt1, and the specific membrane environment cooperate to regulate fusion pore dynamics, which plays an important role in controlling the dose of neurotransmitter release, thereby impacting the signal transduction between neurons.

## Materials and methods

### Protein expression, purification, and labeling

VAMP-2, SNAP-25A, Vps (1–96), MSP1E3D1, C2AB (A418C), C2AB_F349A (A418C), Syt1, and Syt1 mutants (Syt1_QM, Syt1_QQQ, Syt1_F349A, and Syt1_A418C) were expressed in *Escherichia coli* BL21 (DE3) cells and purified as 6×His-tagged or GST-tagged proteins, as described previously with modifications (Tucker et al., 2004; Bao et al., 2018). In brief, VAMP-2, STX1A, Syt1, and Syt1 mutants were expressed at 20°C in *E. coli* BL21 (DE3) cells (Tsingke Biotechnology, TSC-E01). Bacterial pellets were resuspended (∼10 ml per liter of culture) in resuspension buffer (25 mm HEPES–NaOH, pH 7.4, 400 mm NaCl, and 1 mm TCEP). Protease inhibitor (1 mm PMSF) and DNase I (Sigma, 10 μg/ml) were added, and the samples were sonicated on ice for 3 sec (50% duty cycle) twice. n-Dodecyl-β-D-maltoside was added to 2% (*w*/*v*) and incubated overnight with rotation at 4°C before centrifugation of the cell lysate at 35000 rpm for 30 mins in a T-647.5 rotor (Thermo). The supernatant was then incubated for 1–2 h at 4°C with Ni-NTA agarose (Qiagen) or glutathione-agarose (Smart-Lifesciences) for STX1A equilibrated in resuspension buffer. For VAMP-2 or Syt1 and its mutants, agarose beads were washed with buffer I (25 mm HEPES–NaOH, pH 7.4, 400 mm NaCl, 20 mm imidazole, 10% glycerol, 1 mm TCEP, and 0.8% n-octyl-β-D-glucoside (OG)). Proteins were eluted with elution buffer (25 mm HEPES–NaOH, pH 7.4, 400 mm NaCl, 500 mm imidazole, 10% glycerol, 1 mm TCEP, and 0.8% OG). The 6×His tag on VAMP-2 was cleaved by tobacco etch virus protease at room temperature for 3 h, while the 6×His tag on Syt1 and its mutants remained uncleaved. For STX1A, agarose beads were washed with buffer II (25 mm HEPES–NaOH, pH 7.4, 400 mm NaCl, 10% glycerol, 1 mm TCEP, and 0.8% OG), and the protein was cleaved by thrombin (Sigma) on agarose. Then, the proteins were loaded on the Superdex 200 Increase 10/300 GL column (GE Healthcare) for size exclusion.

For the purification of SNAP-25A, Vps, MSP, C2AB (A418C), and C2AB_F349A (A418C), a similar procedure was used except that all detergents were omitted from buffers. The purified proteins were dialyzed against the buffer (25 mm HEPES–NaOH, pH 7.4, 100 mm NaCl, 10% glycerol, and 1 mm dithiothreitol) and loaded on the Source 15Q column (GE Healthcare) for ion exchange.

For protein labeling of VAMP-2, Syt1, C2AB, and their mutants, the proteins and Sulfo-Cy5 maleimide (Duofluor) were mixed at a molar ratio of 1:2, in the presence of 0.5 mm TCEP, and incubated in the dark at 4°C overnight. The free dyes were removed using two consecutive desalting steps via the Pierce Zeba desalting column. The labeling efficiency of was determined to be 34.5%–42%.

### PM-vesicle reconstitution

PM-vesicles were prepared as described previously with modifications (Kyoung et al., 2013). In brief, 3.32 μmol of lipid mixture was added to a glass tube and evaporated with a gentle stream of argon gas. The lipid mixture consisted of 1% L-α-phosphatidylinositol-4,5-bisphosphate (PIP_2_), 15% 1,2-dioleoyl-sn-glycero-3-phospho-L-serine (DOPS), 20% 1,2-dioleoyl-sn-glycero-3-phosphoethanolamine (DOPE), 44% 1-palmitoyl-2-oleoyl-sn-glycero-3-phosphocholine (POPC), and 20% cholesterol (all from Avanti Polar Lipids). Meanwhile, 16.6 nmol STX1A was mixed with vesicle buffer (25 mm HEPES–NaOH, pH 7.4, and 150 mm NaCl) supplemented with 1 mm TECP and 3% OG. In particular, 25 mm SRB was included in the vesicle buffer for preparing PM-vesicles in the content mixing assay. The lipid film was dissolved with the syntaxin-containing solution by vortexing at a low speed. SNAP-25A solution was added to the glass tube while rapidly vortexing to prevent local concentration gradients. The total volume of the resulting solution should be 4.5 times the total volume of the lipid–STX1A mixture, the molar ratio of SNAP-25A to STX1A should be >3, and the combined volume of vesicle buffer and SNAP-25A solution will dilute OG to its critical micelle concentration. PM-vesicles were then isolated by column CL4B (Cytiva), followed by dialysis against vesicle buffer supplemented with 1 mm TECP overnight at 4°C.

### ND reconstitution

Reconstitution of VAMP-2 onto ND was performed as described previously with modifications (Shi et al., 2013; Bao et al., 2018). POPC and DOPS were mixed at PC: PS molar ratio of 85:15 or 92.5:7.5 (900 nmol in total) and air-dried with argon. The lipid film was further dried in a vacuum pump for at least 1 h. The MSP protein was added at a molar ratio of 1:40 to the lipid mixture. Together with MSP, VAMP-2 was also added at MSP: VAMP-2 ratio of 2:0.2 (ND1), 2:0.4 (ND2), 2:1 (ND3), 2:2 (ND4), or 2:4 (ND5) (Shi et al., 2013; Bao et al., 2016).

To make sv-ND, Syt1 was mixed with VAMP-2 at a molar ratio of 1:4, unless otherwise specified. In brief, VAMP-2, Syt1, and MSP were mixed at the specific ratio in the buffer (25 mm HEPES–NaOH, pH 7.4, 150 mm NaCl, 20 μm EDTA, and 1 mm TCEP) supplemented with 50 mm sodium cholate (Sigma). The protein mixture was used to resuspend the dried lipid film, followed by vortexing for 15 min and incubation at 4°C on a shaker for 3 h. The detergent was removed by BioBeads SM-2 (Bio-Rad). The NDs were purified by the Superdex-200 Increase 10/300 GL column with the buffer (25 mm HEPES–NaOH, pH 7.4, 150 mm NaCl, and 20 μm EDTA).

### Negative-stain TEM

PM-vesicle or ND was applied to a continuous carbon-coated EM grid, which was glow-discharged (PELCO easiGlow) for 45 sec. After 1 min of incubation, the grid was blotted dry with filter paper, stained with 1% uranyl acetate (Electron Microscopy Sciences), and air-dried. The negatively stained specimens were examined on a transmission electron microscope (JEOL1400) and operated under an acceleration voltage of 120 kV.

### Content mixing between v-ND and PM-vesicle

The v-ND and PM-vesicle were mixed at 25°C in vesicle buffer (25 mm HEPES–NaOH, pH 7.4, and 100 mm NaCl). The SRB fluorescence intensity was measured at excitation and emission wavelengths of 535 and 565 nm, respectively, with a Duetta fluorescence spectrophotometer (Horiba). The maximum fluorescence intensity was obtained by adding 0.1% reduced Triton X-100 (Sigma).

### Single-channel membrane fusion electrophysiological experiments

The device used in this experiment to build the phospholipid bilayer is a vertical standard lipid bilayer box, in which a white Delrin sample cup (Cup) with the diameter of 1 cm divides the electrolytic cell into the *cis* side (*-cis*) and *trans* side (*-trans*). The *cis*- and *trans*-chambers were connected through a small hole with the diameter of 150 μm on the wall. Then, 0.2 μl of phospholipid solution was coated on the hole of the sample cup with bubbles to form a lipid membrane. A loop current ∼0 pA can be observed. The thickness of the membrane can be evaluated by the membrane capacitance. Lipid bilayers can be formed spontaneously by pulsing high pressure to break the membrane and slowly pulling the buffer into the *cis*-chamber. After the formation of the planar lipid bilayer, PM-vesicles were added to the *trans*-chamber of the device to spontaneously fuse with the planar bilayer. The SNAREpins were deposited into the BLM by slowly pulling the buffer of the *cis*-chamber several times. To form the fusion pore, v-ND or sv-ND was added to the *cis*-chamber, and the opening and closing of the recording pore were observed at −50 mV.

### Preparation of the SLB

A lipid mixture (5 μmol), composed of 43.9% DOPC, 15% DOPS, 5% 1,2-dioleoyl-sn-glycero-3-phosphoethanolamine-N-[methoxy(polyethylene glycol)-2000] (PEG2000-PE), 15% DOPE, 20% cholesterol, 1% PIP_2_, and 0.1% 1,1′-dioctadecyl-3,3,3′,3′-tetramethylindocarbocyanine perchlorate (DiI) (all from Avanti Polar Lipids), was dried in an argon flow followed by desiccation under vacuum. After 4 h, the lipids were resuspended in vesicle buffer (25 mm HEPES-NaOH, pH 7.4, and 100 mm NaCl) with vortexting for 5 min. After 10 freeze–thaw cycles, the suspension was extruded 31 times through a 50-nm polycarbonate membrane (Whatman Nuclepore, Cytiva) using an Avanti Polar Lipids extruder to produce small unilamellar vesicles. The final vesicle suspension was stored at −80°C. Quartz slides were carefully cleaned by sequential sonication in 5% Alconox, acetone, and 1 M KOH, extensively rinsed with deionized water and Piranha cleaning (a 7:3 mixture of sulfuric acid and hydrogen peroxide), and finally extensively rinsed with deionized water. The SLBwas formed by incubating the small unilamellar vesicles with the slides as described previously (Karatekin and Rothman, 2012).

### Single-molecule membrane binding assay

After the SLB was formed, 40 pM Cy5-labeled C2AB or C2AB_F349A was injected into the flow chamber and incubated for 30 mins at room temperature. After removal of unbound proteins using oxygen scavenger buffer (0.1 mg/ml glucose oxidase, 0.02 mg/ml catalase, 0.4% (*w*/*v*) β-D-glucose, and 0.1% cyclooctatetraene), Cy5-labeled C2AB was excited by red (632 nm) laser light. The number of labeled C2AB fragments that bound to DiI-labeled SLB was counted at an excitation power of 3 mW. After rinsing away oxygen scavenger buffer with vesicle buffer, quantification of C2AB within individual fluorescent spots was accomplished by counting sequential stepwise photobleaching events occurring within a single fluorescent spot. Histograms represent the distribution of the number of photobleaching steps, which corresponds to the number of C2AB fragments per fluorescent spot. The distribution of the observed numbers of labeled fragments per fluorescent spot was fitted to a Poisson distribution, assuming a random binding process. The ‘real’ Poisson distribution function, which includes both labeled and unlabeled C2AB fragments, was calculated as described previously (Lai et al., 2016).

### Single-ND lipid mixing assay

DiI-labeled PM-ND (1 mol%) was immobilized on the PEG-coated quartz surface through a neutravidin–biotin interaction followed by removal of unbound PM-ND with vesicle buffer (25 mm HEPES–NaOH, pH 7.4, and 100 mm NaCl). Subsequently, the surface-tethered PM-ND was incubated with 1% DiD-labeled sv-ND for 1 h, followed by rinsing away unbound sv-ND using oxygen scavenger buffer. While recording the images, we excited the first 20 frames by both green laser (532 nm) and red laser (635 nm) to ensure the co-localization of PM-ND and sv-ND. We then switched to green laser (532 nm) to identify the pairing of PM-ND and sv-ND and analyze the FRET efficiency. We excited the last frames by red laser (635 nm) to identify sv-ND. The co-localized spots, exhibiting signals in both the acceptor and donor channels, were then selected and subjected to analysis. To calculate FRET efficiency, we performed data collection, including background subtraction and cross-talk correction of the donor signal to the acceptor channel. From the selected spots, the acceptor and donor time traces were analyzed to generate FRET efficiency histograms.

### Data acquisition and processing parameters

Electrophysiological experiments were performed using a 200B patch clamp amplifier and a Digidata 1550B acquisition system (Molecular Devices) to record currents. pCLAMP 10 software was used to obtain single-channel recordings at 10 kHz, and filtering was performed using a low-flux bessel frequency of 5 kHz. All the recordings were performed at room temperature in a clean room.

### Data analysis

All single-channel data were filtered at 1 kHz and analyzed using Clampfit 10.7, Origin 2021 (OriginLab), and GraphPad Prism 8.

### Data availability

All the data that support the findings of this study are available upon request from the corresponding author.

## Supplementary Material

mjae011_Supplemental_File

## Notes

**Author contributions:** Kaiju Li, Kaiyu Li, J.G., and Y.L. designed the experiments. Kaiyu Li, J.F., Y.X., L.C., and H.L. carried out protein expression, purification, and labeling. Kaiyu Li and X.Z. carried out PM-vesicle reconstitution. Kaiyu Li carried out ND reconstitution, negative-stain TEM, and content mixing experiments. Kaiju Li, C.T., M.L., and Y.Z. performed electrophysiological experiments. J.F. performed the single-molecule membrane binding assay and single-ND lipid mixing experiments. Kaiju Li, Kaiyu Li, J.F., and C.T. analyzed the data. Kaiju Li, Kaiyu Li, J.F., J.G., and Y.L. drafted the manuscript.
