# Supplementary material for: Synergistic regulation of fusion pore opening and dilation by SNARE and synaptotagmin-1
Source: J Mol Cell Biol. 2024 Mar 5;16(4):mjae011. doi: 10.1093/jmcb/mjae011 (PMC11472156; doi:10.1093/jmcb/mjae011)
Supplement: mjae011_Supplemental_File [file mjae011_supplemental_file.pdf]

Supplementary material

Synergistic regulation of fusion pore opening and dilation by SNARE and Synaptotagmin-1

Kaiju Li[1,2,†], Kaiyu Li[1,†], Jiaqi Fan[1,†], Xing Zhang[1], Chengyan Tao[2], Yijuan Xiang[1], Lele Cui[1], Hao Li[1], Minghan Li[2], Yanjing Zhang[2], Jia Geng[2,3,*], and Ying Lai[1,*]

[1] National Clinical Research Center for Geriatrics, State Key Laboratory of Biotherapy and Collaborative Innovation Center of Biotherapy, West China Hospital, Sichuan University, Chengdu 610041, China

[2] Department of Laboratory Medicine, State Key Laboratory of Biotherapy and Cancer Center, West China Hospital, Sichuan University and Collaborative Innovation Center, Chengdu 610041, China

[3] Tianfu Jincheng Laboratory, City of Future Medicine, Chengdu 641400, China

[†] These authors contributed equally to this work.

[*] Correspondence to: Ying Lai, E-mail: ylai@scu.edu.cn; Jia Geng, E-mail: geng.jia@scu.edu.cn

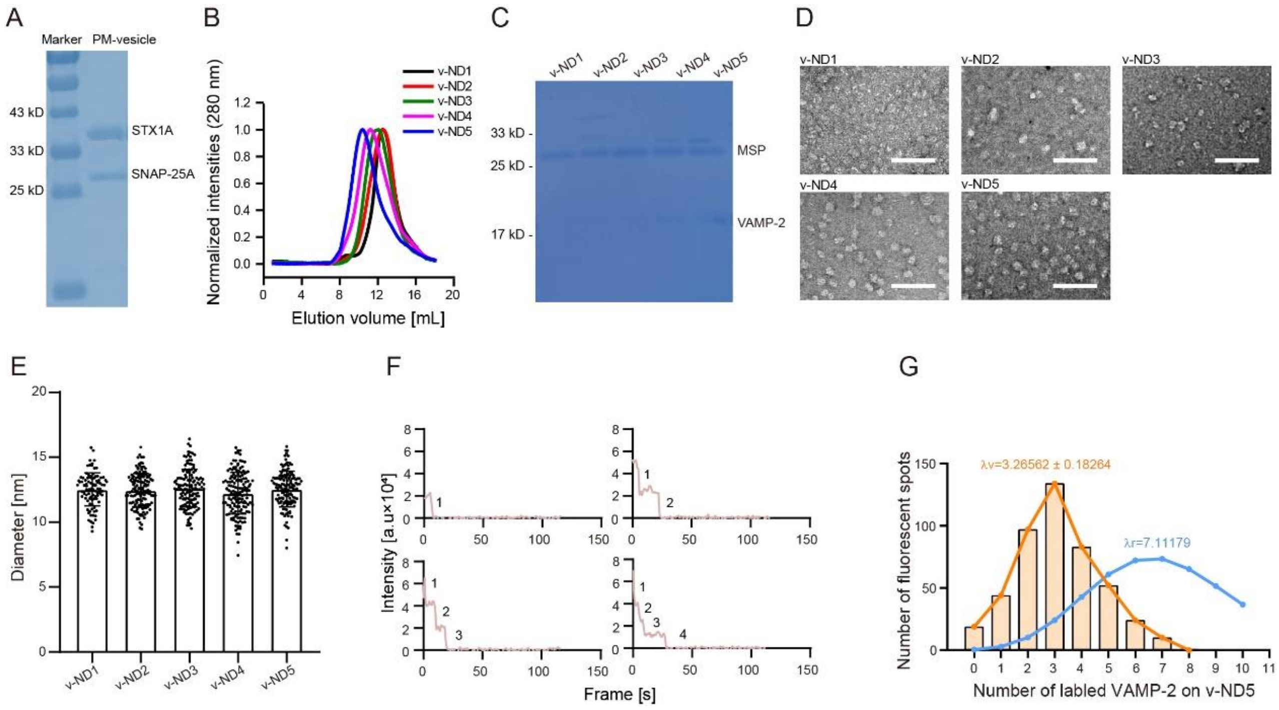

Supplementary Figure S1. PM-vesicle and v-ND reconstitution and characterization. (**A**) SDS-PAGE analysis of PM-vesicle. (**B**) Size exclusion analysis of v-NDs with different copy number of VAMP-2. (**C**) SDS-PAGE analysis of v-NDs. (**D**) Representative negative-stained TEM images of v-NDs. Scale bar, 50 nm. (**E**) The diameter distribution of v-NDs. (**F**) Typical time traces of single-molecule photobleaching steps. (**G**) Distribution of VAMP-2 on v-ND5. The numbers of labeled VAMP-2 could be fit well to Poisson distributions (orange). The blue line is calculated Poisson distributions of all (both labeled and unlabeled) VAMP-2 (Methods). In Panel (**E**), the bar graphs represent as mean ± SEM.

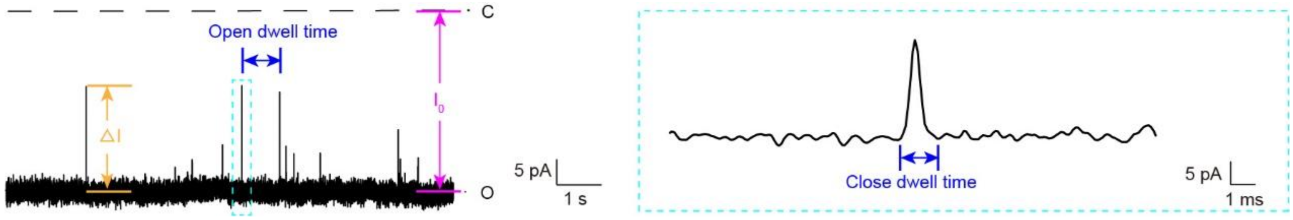

Supplementary Figure S2. Definition of current blocking rate, close dwell time, and open dwell time. Representative open (O) and closed (C) states of a single fusion pore formed. After the stable fusion pore is formed, the ratio of blocking current (ΔI) to open current (I_0) is the current blocking rate; the time within the closed state is defined as the close dwell time, and the time between two closure events is defined as the open dwell time.

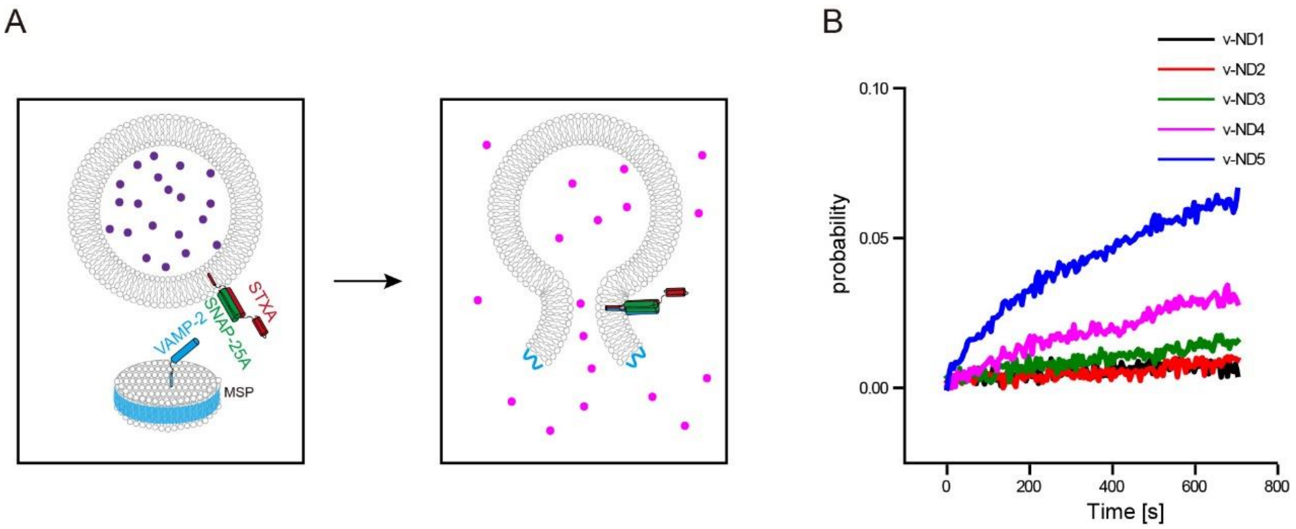

Supplementary Figure S3. Content mixing of v-NDs with PM-vesicle. (**A**) Schematic illustration of content mixing. (**B**) The content releasing probability between PM-vesicle and v-NDs with different copies of VAMP-2.

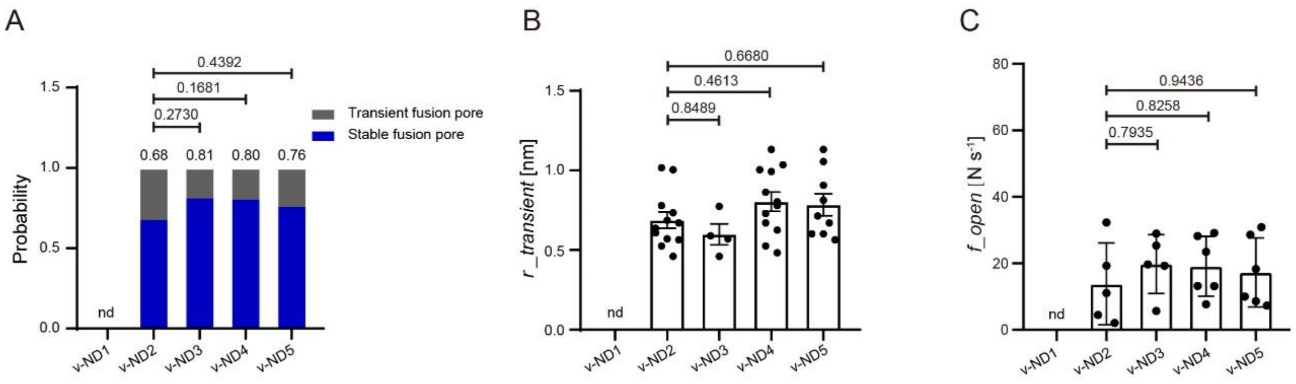

Supplementary Figure S4. Transient fusion pores formation by v-NDs with different copies of VAMP-2. (**A**-**C**) The percentage of stable fusion pores (**A**), the radius of transient fusion pore (**B**) and the open frequency of transient fusion pore (**C**). In panel (**A**), statistical analysis was performed using Pearson's χ^2 analysis test. In panels (**B** and **C**), the bar graphs represent as mean ± SEM and statistical analysis was performed using One-way ANOVA test.

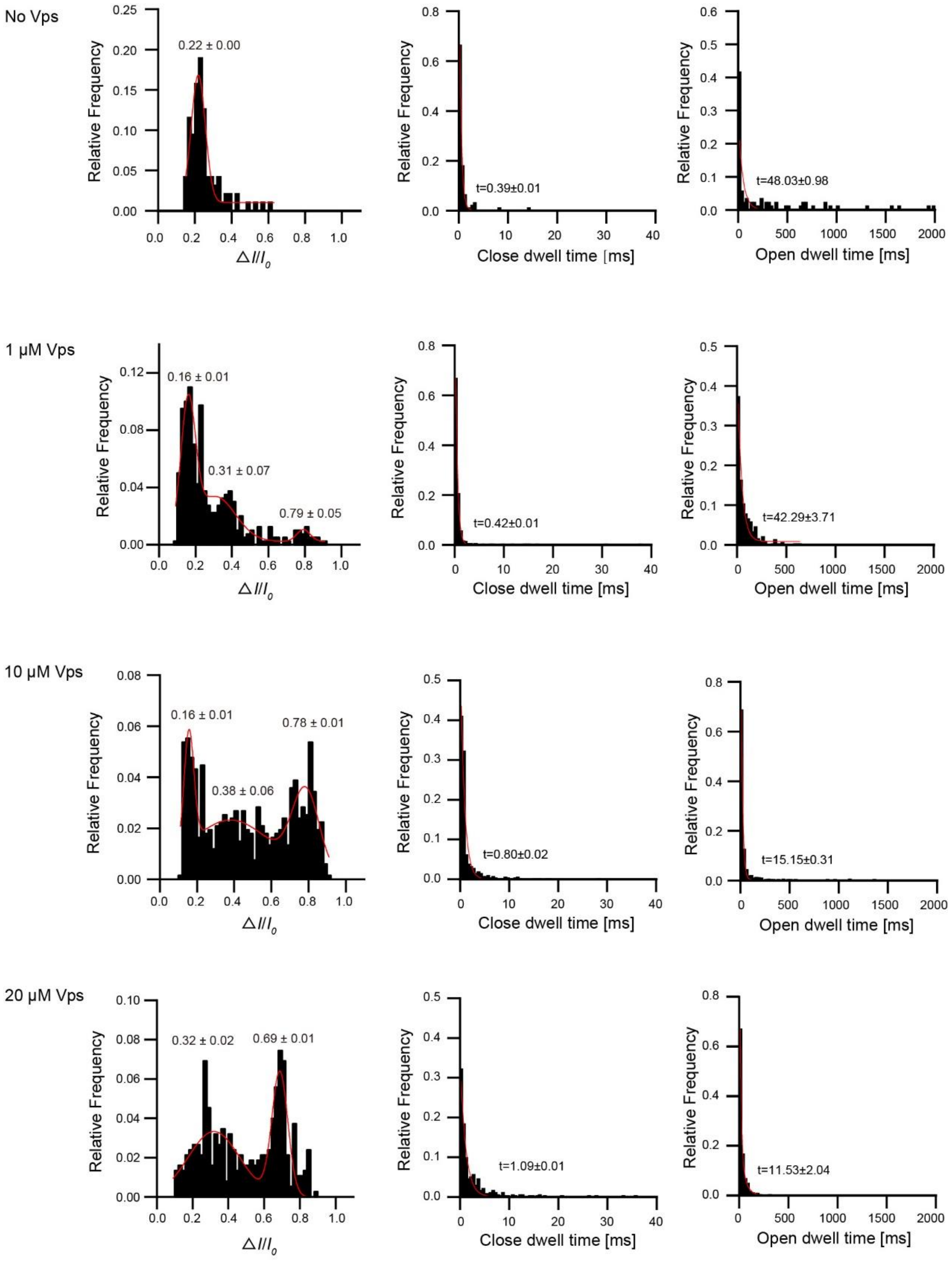

Supplementary Figure S5. Characterization of the effects of Vps on the dynamics of fusion pore opening. The blocking rate ($\Delta I/I_0$), close dwell time and open dwell time of ND5 at different Vps concentration were obtained upon fitting the corresponding histograms with a gaussian function and a single exponential decay function.

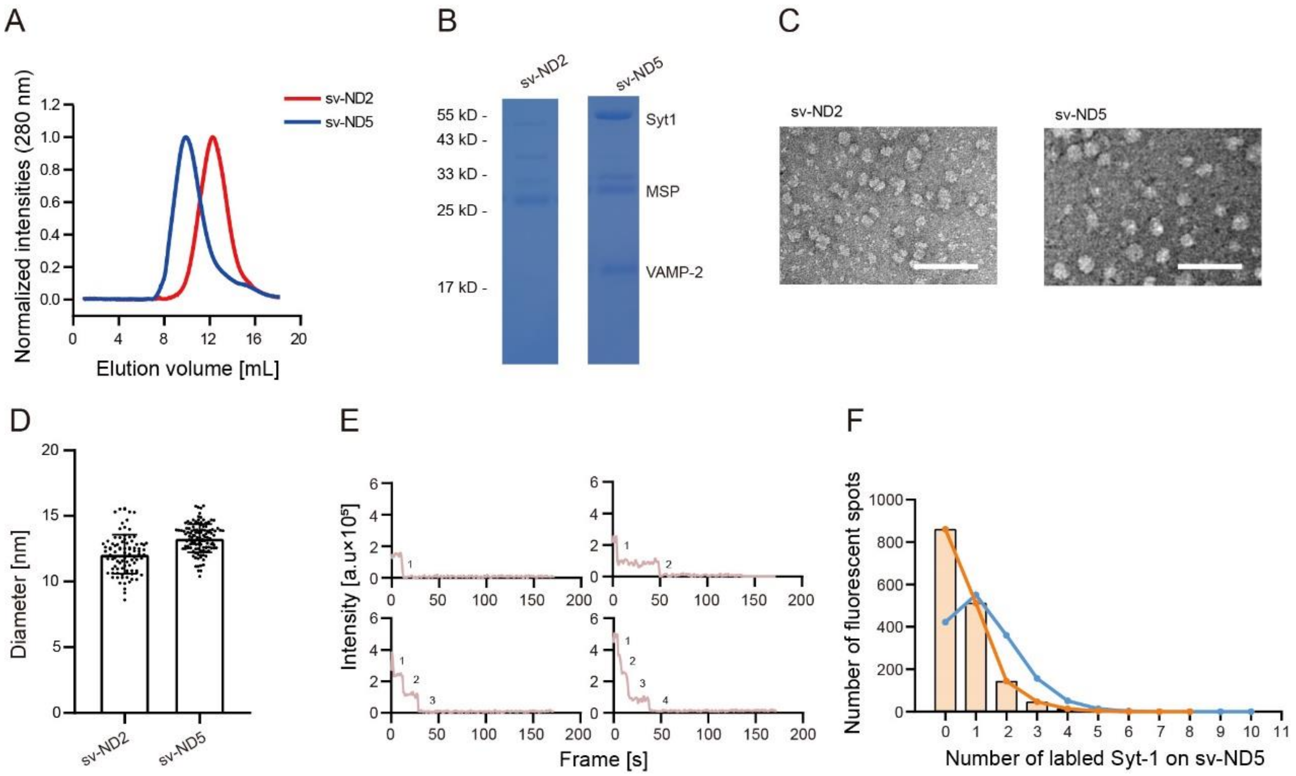

Supplementary Figure S6. sv-ND reconstitution and characterization. (**A**) Size exclusion analysis of sv-ND2 and sv-ND5. (**B**) SDS-PAGE analysis of sv-NDs. (**C**) Representative negative-stained TEM images of sv-NDs. Scale bar, 50 nm. (**D**) The diameter distribution of sv-NDs. (**E**) Typical traces of single-molecule photobleaching steps. (**F**) Distribution of Syt1 (left) and VAMP-2 (right) on sv-ND5. The numbers of labeled Syt1 and VAMP-2 could be fit well to Poisson distributions (orange). The blue line is calculated Poisson distributions of all (both labeled and unlabeled) Syt1 and VAMP-2 (Methods). In panel (**D**), the bar graphs represent as mean ± SEM.

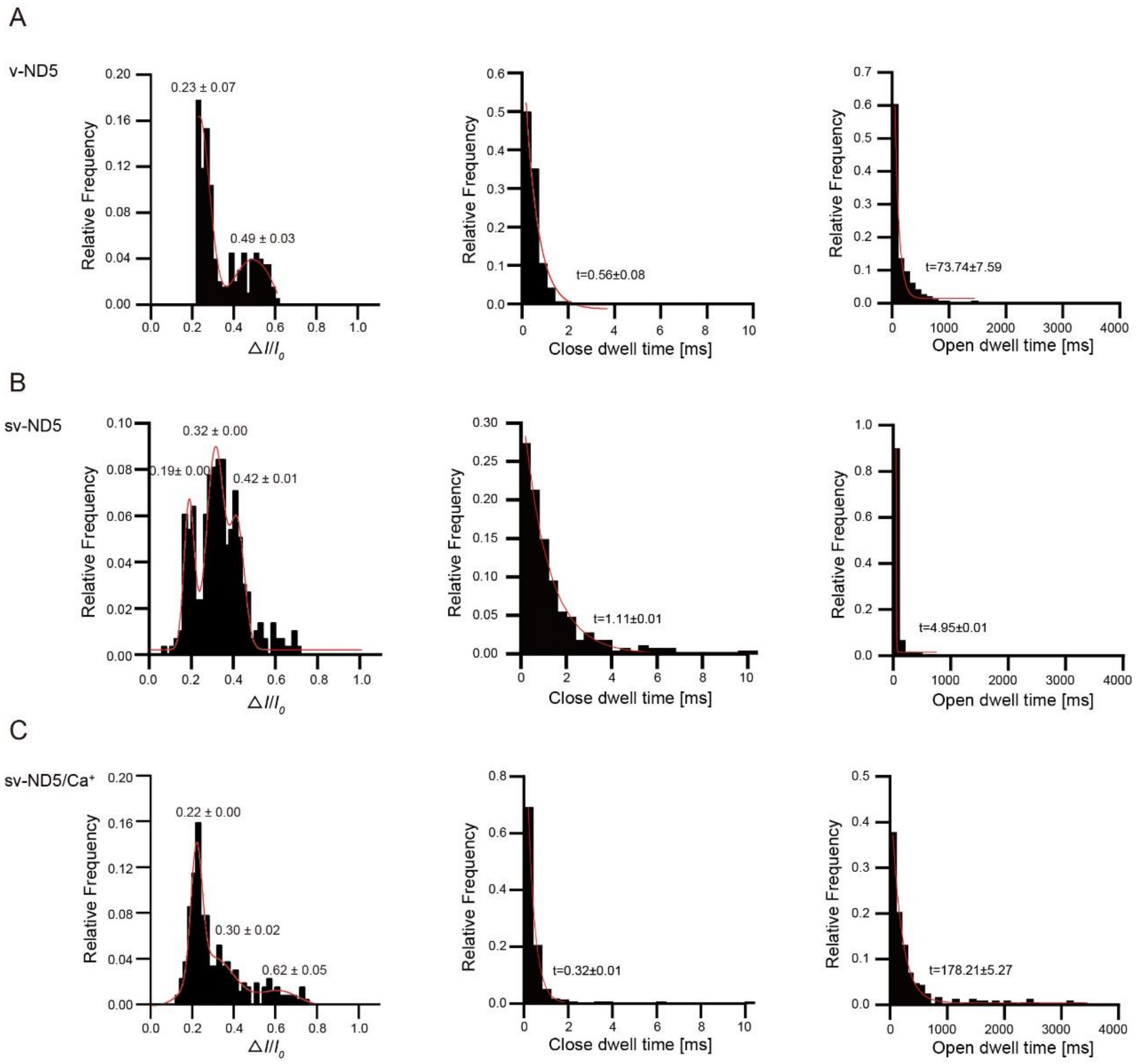

Supplementary Figure S7. Characterization of the effects of Syt1 and Ca^{2+} on dynamics of fusion pore opening. (**A**-**C**) The blocking rate ($\Delta I/I_0$), close dwell time and open dwell time of fusion pore opening by v-ND5 (**A**), sv-ND5 (**B**) and sv-ND5/Ca^{2+} (**C**). The corresponding histograms were fitted with a gaussian function or a single exponential decay function, and the values of blocking rate, close dwell time and open dwell time were indicated in the histograms.

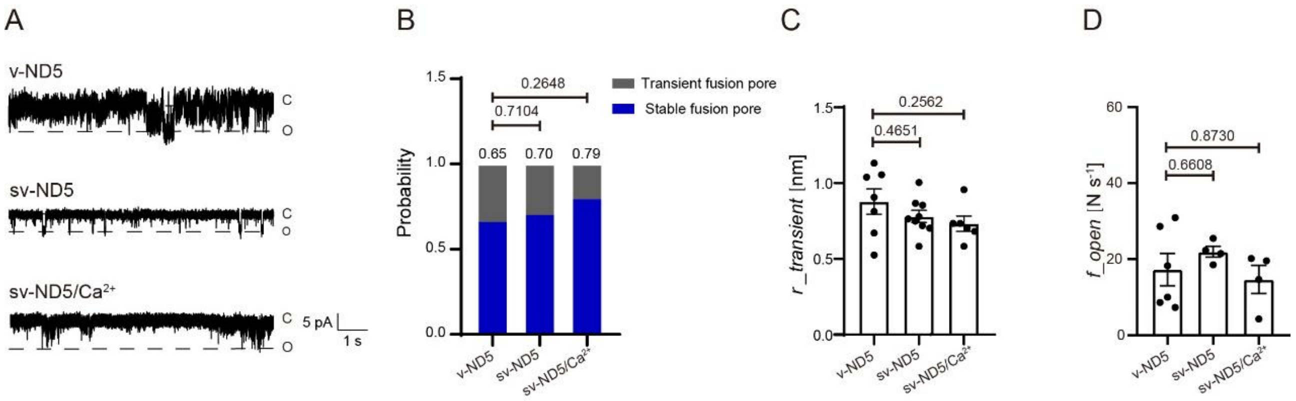

Supplementary Figure S8. Transient fusion pores formation mediated by Syt1 and Syt1/Ca^{2+}. (**A**) Typical traces of transient fusion pore at specified condition, closed (C) and open (O) states are indicated. (**B**-**D**) The percentage of stable fusion pores (**B**), the radius of transient fusion pore (**C**) and the open frequency of transient fusion pore (**D**) formed by v-ND5, sv-ND5 and sv-ND5/Ca^{2+}. In panel (**A**), statistical analysis was performed using Pearson's $\chi 2$ analysis test. In Panels (**C** and **D**), the bar graphs represent as mean ±SEM and statistical analysis was performed using One-way ANOVA test.

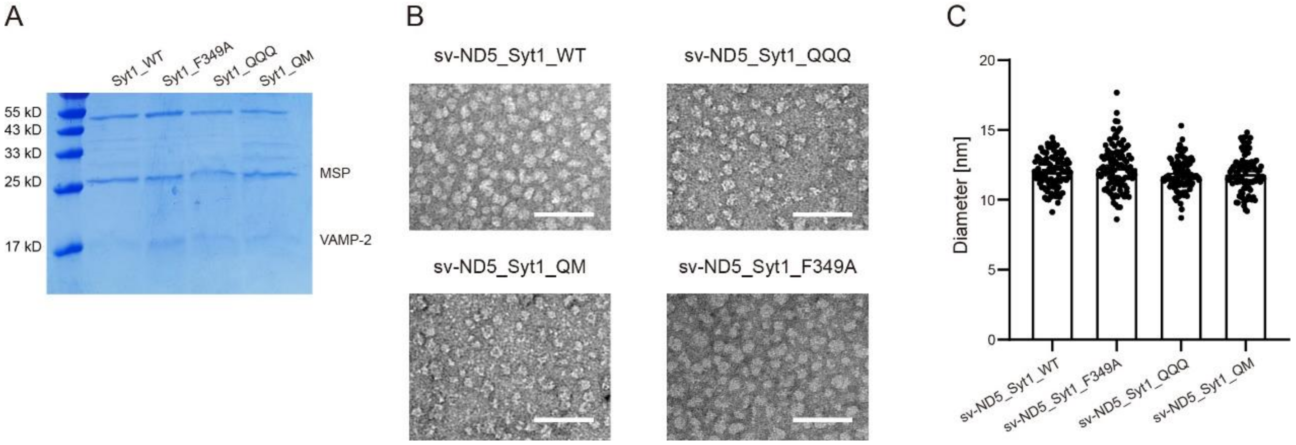

Supplementary Figure S9. Reconstitution and characterization of sv-ND5 with Syt1_WT and its mutants. (**A**) SDS-PAGE analysis of sv-ND5s with Syt1_WT and its mutants. (**B**) Representative negative-stained TEM images of sv-ND5 with Syt1_WT and its mutants. Scale bar, 50 nm. (**C**) The diameter distribution of sv-NDs. In panel (**C**), the bar graphs represent as mean ±SEM.

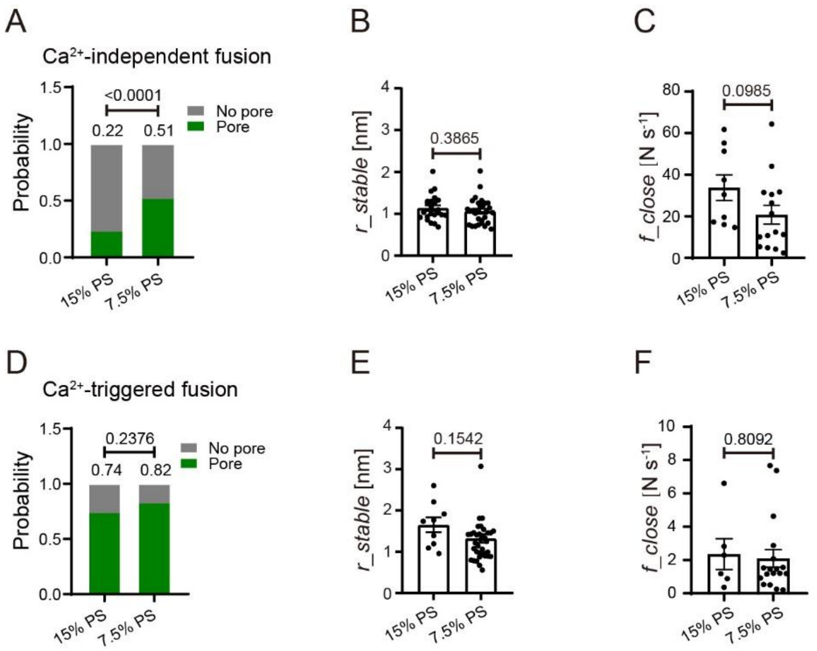

Supplementary Figure S10. Effects of PS concentration on fusion pores opening. (**A**-**C**) The percentage of occurrence of fusion pore formation (**A**), the radius of stable fusion pore (**B**) and the close frequency of stable fusion pore (**C**) with different PS concentrations on sv-ND5 in the absence of Ca^{2+}. (**D**-**F**) The percentage of occurrence of fusion pore formation (**D**), the radius of stable fusion pore (**E**) and the close frequency of stable fusion pore (**F**) with different PS concentrations on sv-ND5 in the presence of Ca^{2+}. In panels (**A** and **D**), statistical analysis was performed using Pearson's $\chi 2$ analysis test. In panels (**B**, **C**, **E** and **F**), the bar graphs represent as mean ± SEM and statistical analysis was performed using student's t-test.

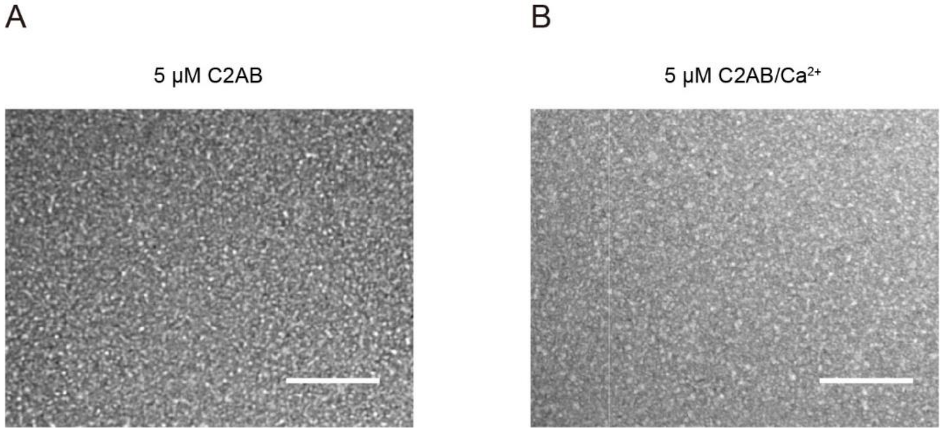

Supplementary Figure S11. Negative-stained TEM image of C2AB. (**A** and **B**) Representative negative-stained TEM image of 5 μM C2AB (**A**) and 5 μM C2AB/Ca^{2+} (**B**). Scale bar, 100 nm.

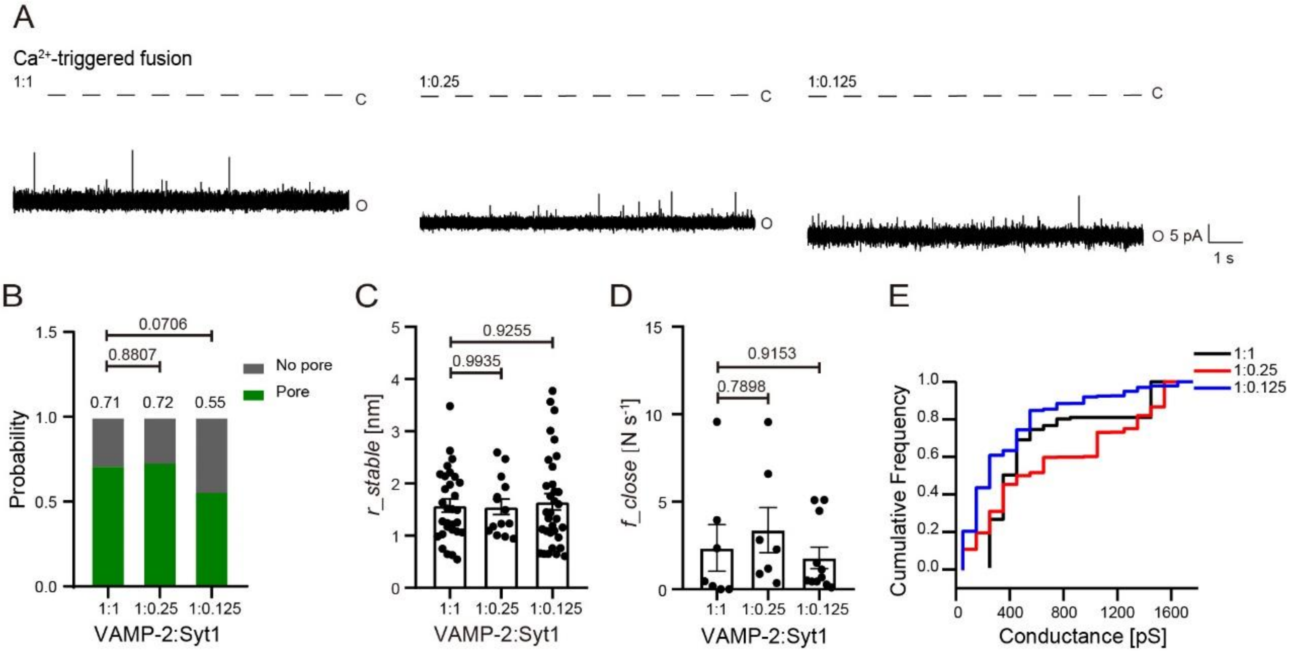

Supplementary Figure S12. The density of Syt1 on ND had little effect on Ca^{2+}-triggered fusion pore opening. (**A**-**E**) Typical traces of fusion pore (**A**), the percentage of occurrence of fusion pore formation (**B**), the radius of stable fusion pore (**C**), the close frequency of stable fusion pore (**D**) and representative cumulative conductance distribution of stable fusion pore (**E**) on sv-ND5 with different VAMP-2/Syt1 ratio in presence of Ca^{2+}. In panel (**B**), statistical analysis was performed using Pearson's $\chi 2$ analysis test. In panels (**C** and **D**), the bar graphs represent as mean ± SEM and statistical analysis was performed using One-way ANOVA test.
